# The Usefulness of Lordosis Load Test and Urinary Biochemistry in the Diagnosis of Orthostatic Proteinuria

**DOI:** 10.7759/cureus.71800

**Published:** 2024-10-18

**Authors:** Yuko Tasaki, Mari Yamamiya, Ria Kasahara, Akane Iwasaki, Takafumi Fukuda, Misato Obata, Mari Nakano, Mika Inoue, Shinobu Sakazume, Kazuhide Ohta

**Affiliations:** 1 Department of Pediatrics, National Hospital Organization (NHO) Kanazawa Medical Center, Kanazawa, JPN

**Keywords:** chronic glomerulonephritis, congenital anomalies of the kidney and urinary tract (cakut), lordosis load test, orthostatic proteinuria, urinary biochemistry

## Abstract

Introduction

Renal disease is commonly suspected in patients with proteinuria. Renal biopsy might be considered based on the patient’s clinical history and the results of diagnostic tests. However, as orthostatic proteinuria is benign and requires no treatment, it is important to obtain a diagnosis without renal biopsy whenever possible. Therefore, up to now, for the diagnosis of orthostatic proteinuria, in addition to resting urinalysis evaluation (disappearance of proteinuria), we have performed the lordosis load test and urine biochemistry of the samples showing peak proteinuria in a lordosis load test.

Method

We retrospectively enrolled all patients who visited the pediatric department and underwent the lordosis load test at Kanazawa Medical Center between 2011 and 2020. In the present study, samples with the highest concentrations of protein after the lordosis load test were subjected to general urinary biochemistry and urinary sediment analysis. Patients were followed up with the lordosis load test for several years.

Results

Enrolled in the study were 68 patients with OP. The mean age at the time of diagnosis was 11.5 years (range, six to 16 years). General urinary tests, urinary sediment, and urinary biochemistry including N-acetyl-beta-D-glucosaminidase (NAG), alpha1-microglobulin(α1MG), and beta 2-microglobulin (β2MG) were normal in all patients with orthostatic proteinuria except one case who was a premature baby.

Conclusion

If proteinuria disappears after two hours of rest, and urinary biochemistry of the samples showing peak proteinuria in the lordosis load test is normal, orthostatic proteinuria can be diagnosed more accurately.

## Introduction

Proteinuria is an important prognostic marker of kidney disease, but benign proteinuria is also common. Orthostatic proteinuria (OP) is a typical example of this condition. OP is benign proteinuria that does not require treatment [1]. It has a peak incidence in adolescents and is rare after 30 years of age [2,3]. It has been reported in a limited number of studies that the prevalence of OP in children and adolescents ranges between 1% and 5%, respectively [2,4]. In Japan, urine testing is performed as part of the school health examination system. In these school urinary screening tests, OP accounts for a large proportion of those who are positive for proteinuria alone. One of the reasons for the large number of OP cases is the urinary collection method. In urine testing, it is important to empty the bladder completely before going to bed and collect urine immediately after waking up. If this is not possible, OP can be diagnosed by observing changes in urinary protein during lordosis loading and resting in an outpatient setting. In Japan, the lordosis load test is often used to diagnose OP [5]. We administer the lordosis load test with the consent of the parents and the children of the subjects.

While there may be many opinions on the status of OP, many OPs are thought to be most likely caused by stagnation of the renal veins [6-9]. However, few studies have evaluated the underlying pathological condition. Especially, it is said to be the same condition as Nutcracker syndrome, but this has not been clearly elucidated. Subclinical nephritis (chronic fixed-stage nephritis) may be mixed with conditions considered OP. Proteinuria in the lordosis test is not specific, because it is also seen in patients with chronic nephritis when viewed as the total amount of excreted urinary protein [5].

Therefore, we perform urinary biochemistry tests including N-acetyl-beta-D-glucosaminidase (NAG), alpha 1-microglobulin (α1MG), beta 2-microglobulin (β2MG), and proteinuria concentration, in addition to the lordosis load test, in pediatric patients with suspected OP. We retrospectively investigated the association between urinary biochemistry test results and proteinuria in patients with OP who visited our institution in the last 10 years.

This article was previously posted to the “Research Square” preprint server on August 16, 2023.

## Materials and methods

Participants and samples

In our department, in children who visit with the chief complaint of proteinuria, if urine protein is negative in the first urine in the early morning and positive in outpatient urine, OP is suspected, and the next test is recommended. We perform the lordosis load test in patients in whom it is possible to rule out congenital anomalies of the kidney and urinary tract (CAKUT) or obvious renal disease by blood tests and abdominal ultrasound examination.

We retrospectively enrolled all patients who visited the pediatric department and underwent the lordosis load test at Kanazawa Medical Center from 2011 to 2020. We reviewed the following data from patients’ medical records: sex, age at the time of the examination, and past medical history. Patients with a history of renal disease, such as chronic glomerulonephritis, were excluded.

**Figure 1:**
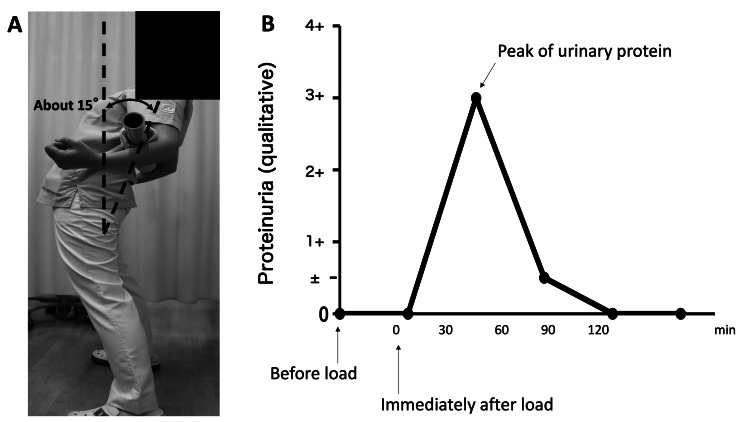
Lordosis load test. (A) Position under lordosis load. (B) A typical example of changes in proteinuria (qualitative) is the lordosis load test. This case is a 12-year-old girl who was diagnosed with OP. OP - Orthostatic proteinuria

After collecting urine just before the test, the patient drinks a cup of water and then holds a lordotic posture (approximately 15 degrees) for five minutes while standing (Figure 1A). A urine sample is collected at the end of this time, and the patient then rests. Further samples are collected at 30-minute intervals over the next 120 minutes. In the present study, samples with the highest concentrations of protein after the lordosis load test were subjected to general urinary biochemistry and urinary sediment analysis. Patients were followed up with the lordosis load test for several years (until two or three years later).

Measurement method

Urinary NAG levels were determined by enzymatic assay (N-assay L NAG; Nittobo Medical Co., Ltd., Fukushima, Japan). Urinary α1-MG and β2-MG levels were determined by latex turbidimetric immunoassay (LZ test “Eiken” α1-M; Eiken Chemical Co., Ltd., Tokyo, Japan; and LZ test “Eiken” β2-M; Eiken Chemical Co., Ltd., respectively). Urinary creatinine levels were determined by enzymatic assay (Aqua-auto Kainos CRE-III plus Test Kit; KAINOS Laboratories, Inc., Tokyo, Japan). All measurement results were corrected for urinary creatinine levels.

Representative case 

A representative case of OP seen at our hospital is presented. A 12-year-old girl visited our hospital with the chief complaint of proteinuria found on school screening. Her protein in early morning urine was negative, and it was positive in outpatient urine. Abdominal ultrasound showed no abnormalities. Blood tests showed no abnormal findings. Based on these results, OP was suspected, and a load test was conducted. The results are shown in Figure 1B. Proteinuria immediately before and after the test was negative, but proteinuria 30 min after the test was 3+ and became negative 90 min after the test. The protein-to-creatinine (Pro/Cr) ratio in the urine sample at 30 min, which is the peak of proteinuria, was 8.6, and urine biochemistry tests were all normal.

Statistical analysis

Data values are expressed as means ± SD. We analyzed the correlation between urinary protein and biochemistry. Statistical analyses were conducted using Excel statistical software to perform linear regression analysis.

Ethics

This study was approved by the Kanazawa Medical Center Ethics Committee (protocol number: R04-047). Patients retained the right to opt-out.

## Results

Enrolled in the study were 68 patients with OP (23 males, 45 (66.2%) females). Some patients underwent repeated lordosis load tests, and 168 samples were collected and analyzed. The mean age at the time of diagnosis was 11.5 years (range, six to 16 years). Most patients were elementary or junior high school students and were followed up for several years: 11 for two years, 11 for three years, six for four years, and 12 for over five years. 

Peak proteinuria values were found immediately after the lordosis load test in 67 (39.9%) samples, 30 minutes after loading in 99 (58.9%) samples, and 60 minutes after loading in two samples. The mean peak concentration of proteinuria was 319 ± 296 mg/dL. Thus, proteinuria was found to be induced in early phases after the lordosis load test. Urine sediment values were normal in all samples, regardless of the degree of proteinuria. Furthermore, the urinary biochemistry results were as follows: urinary α1-MG, 2.2 ± 1.2 mg/g.Cr (normal value <3.8); β2-MG, 199± 139 μg/g.Cr (normal value <600); and NAG, 5.7± 4.7 IU/g.Cr (normal value <10.0) (Table 1). The above results show that the lordosis load test induces a large amount of proteinuria, but the urinary sediment and urine biochemistry tests were almost normal. Of course, as is involved in the definition of OP, all patients disappeared the proteinuria within two hours after rest.

**Table 1:** Patient demographics and clinical data Urinary biochemistry presents peak data after the lordosis load test.

Data	N (%) or mean (SD)	Normal value
Patients	68	ー
Female	45 (66.2)	ー
Female/male	1.96	ー
Peak proteinuria (mg/dL)	319 (296)	< 30.0
Urinary Pro/Cr	3.5 (3.5)	< 0.15
Urinary α1MG (mg/g.Cr)	2.2 (1.2)	< 3.8
Urinary β2MG (μg/g.Cr)	199 (139)	< 600
Urinary NAG (IU/g.Cr)	5.7 (4.7)	< 10.0

**Figure 2:**
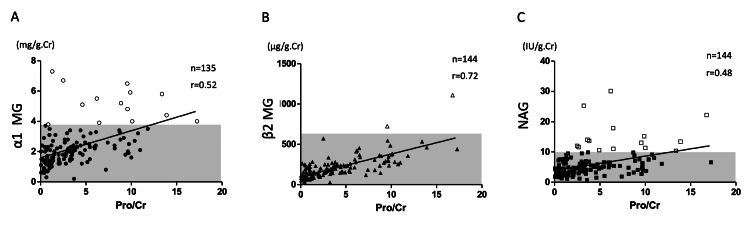
Correlations of urinary protein and biochemistry values. (A) α1MG, (B) β2MG, (C) NAG The shaded areas indicate the normal ranges. The filled markers are normal value data, and values higher than normal range are shown as the open markers. Urinary biochemistry values are within the normal ranges in most patients. The correlation coefficient “r” was determined by Spearman’s rank test (“n” - number of cases).

**Figure 3:**
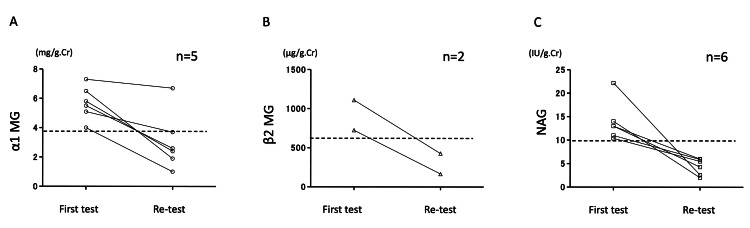
Initial and re-test urinary biochemistry values. (A) α1MG, (B) β2MG, (C) NAG Of the cases with abnormal values, five cases of urinary α1MG (n=5), two cases of urinary β2MG (n=2), and six cases of urinary NAG (n=6) were each retested approximately one year later. Repeat urinary biochemistry results became normalized in all but one of the patients (cases of prematurity). The dashed lines indicate the upper limits of normal for α1MG, β2MG, and NAG, respectively.

As mentioned above, urinary biochemistry values were within the normal ranges in most patients. All urinary biochemistry values and proteinuria were positively correlated (Figures 2A-2C). So, urinary biochemistry values were highest in patients with advanced proteinuria. Urinary biochemistry tests with values above the upper limit of normal were the following: urinary α1-MG 14 samples; β2-MG, two samples; and NAG, 15 samples. Furthermore, changes over time were measured in those who had elevated urine biochemistry results at the time of the initial the lordosis load test approximately one year later. The normalization of urinary biochemistry was confirmed in all but one of the patients (cases of prematurity) by repeating the lordosis test (Figures 3A-3C). Patients with a history of prematurity who presented with this OP pattern were assumed to have some tubular dysfunction present, but this is not confirmed by renal biopsy or other means.

## Discussion

The present study is the first to report correlations between urinary protein and urinary biochemistry results in patients with suspected OP. In OP, proteinuria occurs in the standing position, but not in the resting supine position, and it is found most commonly in young people [10]. The actual prevalence of pediatric OP is uncertain. In the present study, children aged six to 16 years had OP. This was the same age group as reported previously [11]. Most cases of elevated urine protein alone detected at school urine examinations (by dipstick) are OP [12], and the cause is not evident [13]. OP is considered a transient proteinuria that requires no treatment [10]; however, it is difficult to establish the diagnosis. The present study was conducted to test the hypothesis that urinary biochemistry results of urine with a high protein concentration would not be elevated in patients with OP.

Although it is most convenient to compare urinary Pro/Cr at rest and standing, our hospital conducts a lordosis load test to make a diagnosis. Indeed, if early morning urine is negative, no further testing may be necessary. However, it is also true that many children have urinated completely before going to bed on the previous day and have not been able to accurately collect the first urine when they wake up. Therefore, we perform a lordosis load test at an outpatient clinic and use it for the diagnosis of OP. In addition, when comparing two points at rest and standing, even patients with subclinical nephritis may show positive results when standing. We are conducting a lordosis load test and also evaluating urine biochemistry during lordosis load.

The present results showed that a large amount of urinary protein was discharged under loading in many OP patients, but the urinary biochemistry remained at normal levels even at times of peak proteinuria. This finding reconfirms that OP is a benign disease. Urinary biochemistry is useful in the differential diagnosis of other renal diseases. However, the urinary biochemistry values were elevated in the present patients who presented with massive proteinuria. A previous study reported that massive glomerular proteinuria may cause marked U-NAG excretion and moderate urinary U-beta 2M elevation independent of primary renal disease [14]. Therefore, the recovery stage of chronic glomerulonephritis cannot be ruled out in such a case. To investigate this problem, we repeated the lordosis load test and evaluated the results. Following re-examination at follow-up (six months to one year later), all values were normal except for one examination (α1-MG) in one patient. This patient was born prematurely and with an extremely low birth weight. This patient might have an abnormally low number of nephrons rather than OP. Based on the present results, we consider that it would be useful to assess the recovery phase of pure OP and chronic glomerulonephritis with the lordosis stress test and urine biochemistry, as well as follow-up for several years. 

There are some limitations of this study. There is a limitation because OP is determined based solely on the results of the lordosis load test and history of kidney disease, etc. Furthermore, occult blood was observed in some patients, but a change in its occurrence was not evaluated during follow-up. In addition, the method used to diagnose OP differs from that used in Europe and the United States, in which proteinuria is assessed at rest by dipstick, without a lordosis load test. Incorporating a lordosis load test is more complicated than the method of confirming urinary protein disappearance by rest alone. It has been reported that people with lower BMI show persistent OP as opposed to those with higher BMI [15]. We were unable to evaluate the BMI of the patients in this study. However, we consider that, even with these limitations, this report is worthwhile because it is the only report evaluating urine biochemistry in a large number of OP cases.

## Conclusions

When diagnosing benign OP, the disappearance of proteinuria with rest alone is not sufficient. Confirmation of an increase in proteinuria by the lordosis load test and normal urinary biochemistry in the urine sample will aid in a more accurate diagnosis. Evaluation of urinary biochemistry, including repeat lordosis load testing, can also help differentiate patients with stable chronic glomerulonephritis from OP.
